# *Teucrium polium* Extract Enhances the Anti-Angiogenic Effect of Tranilast in a Three-Dimensional Fibrin Matrix Model

**DOI:** 10.31557/APJCP.2021.22.8.2471

**Published:** 2021-08

**Authors:** Fatemeh Sheikhbahaei, Seyed Noureddin Nematollahi-Mahani, Mozafar Khazaei, Mohammad Rasool Khazaei, Saeed Khazayel

**Affiliations:** 1 *Department of Anatomy, Afzalipour School of Medicine, Kerman University of Medical Sciences, Kerman, Iran. *; 2 *Physiology Research Center, Institute of Neuropharmacology, Kerman University of Medical Sciences, Kerman, Iran. *; 3 *Fertility and Infertility Research Center, Health Technology Institute, Kermanshah University of Medical Sciences, Kermanshah, Iran. *

**Keywords:** Angiogenesis inhibitors, Teucrium polium, tranilast, HUVEC, aortic rings

## Abstract

**Objective:**

Angiogenesis plays a dominant role in many pathophysiologic disorders, including cancer. Tranilast, which is an anti-fibrotic drug, is also suggested as an anti-angiogenesis agent. As Teucrium polium (TP) is known as an herbal medicine with antitumor properties, this study aimed to investigate the effects of TP and Tranilast on human umbilical vein endothelial cells (HUVECs), in vitro model of angiogenesis, as well as rat’s aortic ring ex vivo model.

**Methods:**

In this study, The HUVECs were treated with various doses of TP and Tranilast each one alone or in combination together. Cell survival test, aortic ring ex-vivo assay, and evaluating mRNA expressions of VEGFA and TGF-β ligands and receptors were performed.

**Results:**

The survival rate of HUVECs has significantly (p<0.05) reduced by TP and Tranilast. The combination of both TP and Tranilast significantly reduced cell viability as compared to the administration of TP or Tranilast alone. As well, the treatment of HUVECs with TP and/or Tranilast significantly (p<0.05) decreased TGF-β1, TGF-β 2, TGF-βRI, and TGF-βRII mRNA expression levels, but not the expression of TGF-β3 and TGF-βRIII in the TP-treated cells. Image analysis showed that TP and/or Tranilast inhibited vascular growth in the aortic ring assay.

**Conclusion:**

Our results strongly support the anti-angiogenic effects of the TP and Tranilast combination on both in vitro and ex vivo models of angiogenesis. However, further investigations in in vivo models and human studies are needed before human use.

## Introduction

Angiogenesis inhibition, as a promising approach for cancer therapies, is rather essential for both tumor’s development and maintenance (Scappaticci, 2002). It is crucial to investigate numerous biological functions such as physiologic regeneration in ovulation; embryonic development; and pathologic changes such as diabetic retinopathy, rheumatoid arthritis, and solid cancers growth (Hanahan and Folkman, 1996). Angiogenesis is a well-organized series of events, which are usually started by the destruction in tissues and hypoxia. Following angiogenic factors’ activation, it was shown that endothelial cells migrate, proliferate, stabilize, and finally expedite the angiogenic processes (Nishida et al., 2006). 

Angiogenesis mostly depends on the balance between the activation of inhibitory and stimulatory molecules that could stop or initiate this incident (Sheikhbahaei et al., 2018). Accordingly, this is regulated by various growth factors, chemokines, and cytokines. Moreover, different proteins have been introduced as angiogenic activators, including vascular endothelial growth factor A (VEGFA), basic fibroblast growth factor (bFGF), transforming growth factor (TGF)-α, TGF-β, hepatocyte growth factor, and epidermal growth factor. Among these, VEGFA and TGF- β1 were observed to play a prominent role in the regulation of angiogenesis (Ferrari et al., 2009). In this regard, VEGFA has been proved as a pivotal regulator of angiogenic process. Proliferation, survival, and migration are directed by this factor in endothelial cells (Otrock et al., 2007). In addition, VEGFA also plays a principle role in malignant tumors growth through neovascularization, and it can be up regulated by oncogenes expression (Carmeliet, 2005). Hence, the inhibition of VEGFA has been considered for the suppression of angiogenesis as well as the treatment of solid tumor malignancies (Ferrari et al., 2006). It was shown that Transforming growth factor- β (TGF- β) could regulate several biological processes such as proliferation, apoptosis, migration, tumorigenesis, and angiogenesis. Additionally, there are three isoforms of TGF- β (including β 1, β2, and β 3) and three TGF- β s receptors, including I, II, and III (Darakhshan and Pour, 2015). It was reported that TGF- βs, which are produced by tumor cells, could stimulate tumorigenesis through inducing angiogenesis (Massagué, 2008). Several previous studies have proved the importance of TGF-β signaling pathway in angiogenesis (Goumans et al., 2003). It is obvious that both VEGFA and TGF- βs inhibition can be proposed as a decisive novel therapy for solid tumors. 

Tranilast (N- [3, 4-dimethoxycinnamonyl]-anthranilic acid) was firstly introduced in 1976 as a drug used for the treatment of allergic disorders, hypertrophic scars, and keloids. Tranilast inhibits fibroblasts proliferation and subsequently suppresses collagen deposition. Aside from these, Tranilast was found to inhibit the release of TGF-β from fibroblasts (Sheikhbahaei et al., 2018). Antitumor effects of this agent have also been reported in some cell lines (Platten et al., 2001; Izumi et al., 2009). Moreover, it could block cell cycle progression of both MDA-MB- 231 and BT-474 cells (Nie et al., 1997). The Antiangiogenic effects of Tranilast on human dermal microvascular endothelial cells was found to be through the inhibition of the proliferation, tube formation, and expression of VEGFA (Isaji et al., 1997).

Combination therapies with herbal extracts have recently received great interest as suitable solutions to overcome drug resistance and also to increase the drug potency (Nathan and Scholten, 1999). Teucrium polium (TP) belongs to the Lamaceae family, which has long been used in traditional medicine due to its pharmacological properties. Accordingly, it has antipyretic, antioxidant, antibacterial, anti-inflammatory, hypolipidemic and antiseptic properties. Moreover, the anticancer properties of TP on a variety of cancer cell lines have been studied (Khazaei et al., 2018). The antitumor potentials of the different fractions of TP have been investigated as well, indicating that petroleum ether fraction is the most potent fraction on U87 cells (Nematollahi-Mahani et al., 2012). As the inhibition of angiogenesis is currently considered for both the tumor growth inhibition and metastasis, our previous study also indicated that the combination of TP and Tranilast could inhibit angiogenesis in HUVECs, leading to the reduced cell viability, the increased apoptosis, and the decreased migration capacity of these cells. Therefore, this research aimed to investigate wheather the Tranilast and TP combination could affect endothelial cells spurting in a three-dimensional aortic ring assay in an in vivo model of angiogenesis, and to understand the probable molecular changes in the angiogenesis pathway.

## Materials and Methods

The ethics committee of Kermanshah University of Medical Sciences approved this study. In addition, the studied animals received care as recommended by the Ethics Research Committee of the Kermanshah University of Medical Sciences (EC/KNRC/90-4) in terms of the internationally accepted principles for laboratory animal use and care, as proposed in the European Community guidelines (EEC Directive of 1986; 86/609/EEC).

*Material*

*Extraction of the Plant Material*

The areal parts of TP were collected from Kerman, Iran, and then identified at the Department of Pharmacognosy, faculty of Pharmacy, Kerman University of Medical Sciences. Thereafter, a specimen was deposited at the herbarium of the Kerman Faculty of Pharmacy (Voucher number: 28125). Finally, Methanolic extraction followed by petroleum ether fractionation were conducted as described earlier (Sheikhbahaei et al., 2018).

*Cell line and reagents*

For this in vitro experimental study, HUVECs were purchased from the National Cell Bank (Tehran, Iran). Moreover, the culture media and reagents were obtained from Sigma-Aldrich Chemical Co (St. Louis, MO, USA), unless otherwise stated.

*Cell culture and treatment*

The cells were cultured in T75 flasks containing DMEM/F12 medium supplemented with 10% FBS (complete medium) at 37ºC in a humidified atmosphere of 5% CO2. In our previous study, for dose response, the cells were treated for 24, 48, and 72 h with 25, 50, 100, 200, and 400 μg/ml petroleum ether (PE) fraction of TP, along with 75, 150, 300, 600, and 1200 μM of Tranilast dissolved in DMSO. Subsequently, for the analysis of median effect, the fixed ratio drug combinations were done according to IC_50_ values of TP and Tranilast using GraphPad Prism 5 (GraphPad Software Inc., La Jolla, CA, USA). For performing other tests, concentrations of 100 μg/ml of TP and/or 300 μM of TQ (about their IC_50_s) were chosen. Notably, each treatment was replicated at least three times. 

*Animals*

Male Wistar rats weighing 200±250 g were obtained from animal house of Kermanshah University of Medical Sciences, Kermanshah, Iran. Next, they were maintained under 12:12 hour light/dark cycle and fed with standard pellet chow and watered libitum.

*Methods*

*Viability assay using trypan blue exclusion test*

At this stage, HUVECs were seeded in 24-well plates at a density of 7×10^4^ per well for 24 h. The culture medium was replaced with a fresh DMEM/F12 medium containing various concentrations of both Tranilast and TP. The plates were incubated for 24, 48, and 72h at 37º C in a humidified atmosphere with 5% CO_2_. These cells were then trypsinized and the cell suspension was also mixed with an equal volume of 0.4% trypan blue solution. Thereafter, this mixture was transferred to an improved Neubauer hemacytometer and then analyzed under a light microscope at ×40. Finally, the number of the alive cells (unstained) versus the total number of the cells was calculated as the percentage of viability (Strober, 2001).

*Real time RT-PCR*

The effects of various concentrations of Tranilast and TP on the expression levels of some angiogenic-related genes were analyzed using real-time PCR. Total RNA was prepared from HUVECs using the GeneMATRIX Universal RNA Purification Kit (EurX, Poland), in terms of the manufacturer’s instructions. Afterward, Complementary DNA (cDNA) was synthesized using cDNA synthesis kit (reverse transcription PrimeScriptTM RT Reagent Kit, Takara. Japan) in 20 μl of the reaction mixture in terms of the manufacturer’s instructions. Real-time PCR was then performed using SYBR Premix Ex Taq technology (Takara Bio Inc. Japan) on the Applied Biosystems Real-Time PCR One Step System in triplicate. In this process, ACTB was used as an internal control. As well, the primers were designed using the Bio Edit and BLAST programs. (http://www.ncbi.nlm.nih.gov) After analyzing the obtained data using One Step Software v3.2, the relative expression level of these genes was calculated using two –ΔΔCT formula. Subsequently, the primers were designed using the Bio Edit program, and also a BLAST search (http://www.ncbi.nlm.nih.gov) was performed to confirm the specificity of the selected nucleotide sequences. Correspondingly, the primers are listed in Table 1. 

*Rat aortic ring assay*

The study rats were anaesthetized with intraperitoneal (i.p.) injection of chloral hydrate (350 mg/kg). Thoracic aortae were removed from male rats aged between 8 and 12 weeks old, and were then cut into 1-2 mm rings, carefully. Fibrin scaffold was used for three-dimensional (3D) culturing. Using this method, fibrinogen (3 mg/mL) was dissolved in a M199 medium and then added to each well of a 24-well plate (0.5 mL/well). Afterward, 15 μL of thrombin (Stago) was added to each one of these wells. After the gel formation, aorta rings were embedded in the center of the wells and then covered by an additional 0.5 mL of fibrinogen/thrombin solution. When the first vascular sprouts from the aortic rings appeared, they were treated with 300 μM of Tranilast or 100 μg/ml of TP as well as their combination. Next, the explants were cultured for 10 days at 37ºC in a humidified atmosphere of 5% CO2. Aorta rings were daily observed, and were photographed on both days 0 and 10 using an inverted microscope (AE-31; Motic, Barcelona, Spain). Moreover, angiogenesis was assessed according to the number of the wells using a scale from 0 (no growth) to 1–4 (tissue growth and changes [cell invasion into fibrin matrix] seen in <25%, 26%–50%, 51%–75%, and >75% of the cultured tissues, respectively), showing different stages of growth (Aplin et al., 2008).

*Statistical analysis*

The statistical analyses of this study were conducted by SPSS software, version 16 (SPSS Inc., Chicago, IL, USA) with mean ± SD. One-way analysis of variance ANOVA test was used to determine the significance of differences amongst the different studied groups. All the obtained data were reported as mean ± standard error of mean (SEM). A p-value less than 0.05 was considered as statistically significant.

## Results

*Viability assay*

**Figure 1:**
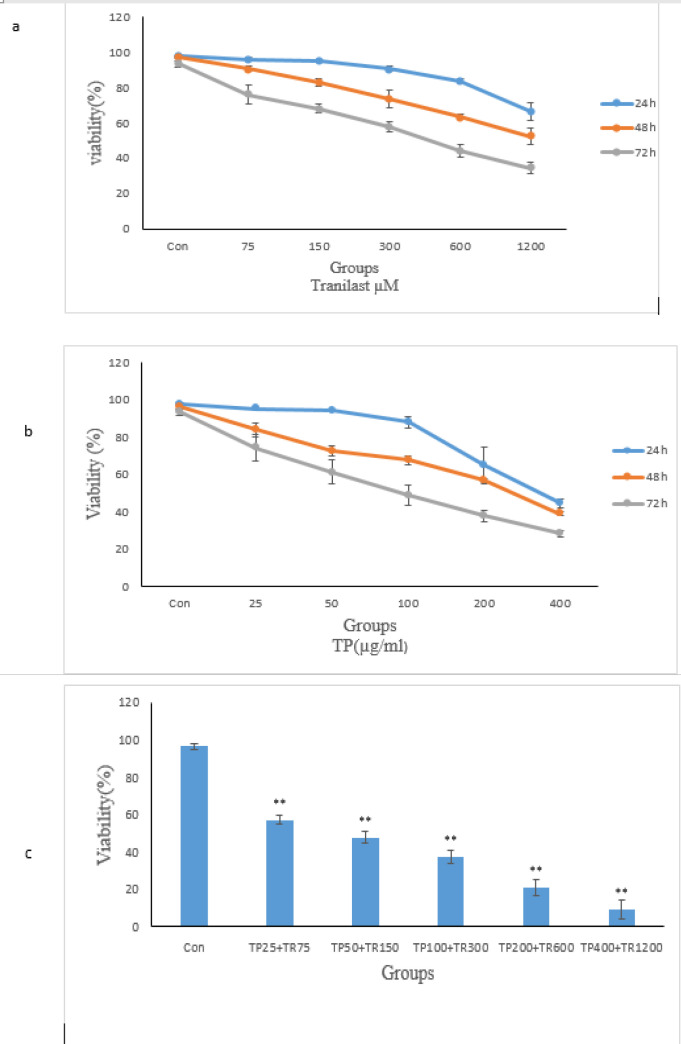
the Effects of the Administrations of *TP* and Tranilast Alone and in Combination on HUVECs Viability Assessed by Trypan Blue Staining. The cells were treated with *TP* and/or Tranilast for 24, 48, and 72h and viability was measured by Trypan blue staining. The control wells were treated with equivalent amount of medium alone. The values are defined as the mean and SE from the triplicated experiments

Viability decreased from the starting dose of 25 μg/ml of TP and dose-dependently continued at either of the time intervals tested. Of note, the highest decrease of the viability was observed at 400 μg/ml and the IC_50_ was calculated to be 107 μg/ml (Figure 1.a). The treatment of the HUVEC cells with the starting dose of tranilast (75 μM) showed no significant effect on the viability rate after 24 and 48 h, but it decreased after 72 h incubation. Thereafter, by IC_50_ the Tranilast dose to 1200 μM, a lower viability rate was observed in HUVECs and the IC_50_ was calculated as 302 μg/ml (Figure 1.b). As well, the combined treatment with TP and Tranilast resulted in a lower viability rate compared to each one of the single treatment protocols (Figure 1.c) (Sheikhbahaei et al., 2018).

*The effects of TP, Tranilast, and their combination on mRNA levels of VEGFA and TGF-β ligands and receptors in HUVECs*

**Figure 2:**
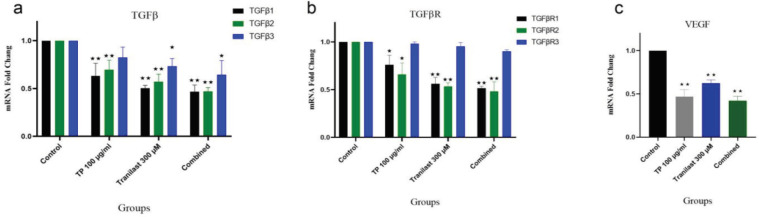
Quantitative RT-PCR Analysis for: a) TGFβ, b) TGFβR, and c) VEGFA genes in HUVECs. Data are expressed as mean ± SEM of three dependent experiments. A significantly difference was observed compared with the control *p < 0.05, **p < 0.001

**Figure 3:**
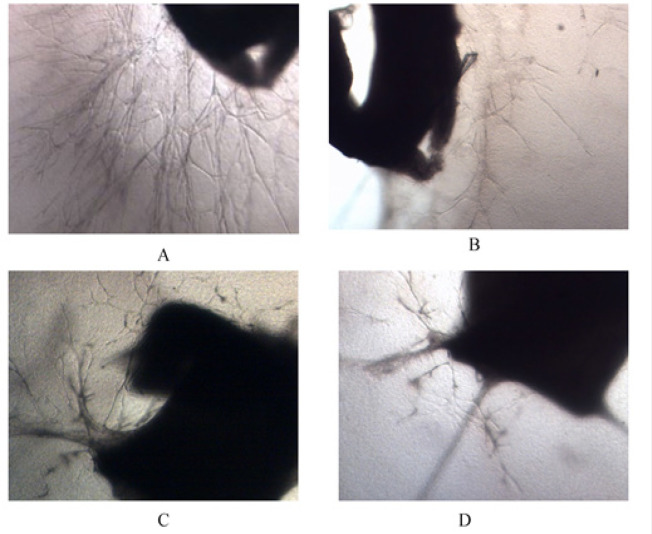
Inverted Micrographs Showing Vessel Outgrowth Raised from the Rat Aorta Rings into Fibrin Matrix on the Day 10 of Culturing (a) the control, (b) 300 μM Tranilast, (c) 100 μg/ml TP, and (d) combined groups

The exposure of HUVECs to both TP and Tranilast alone or in combination for 72 h decreased the expression levels of TGF-β1, TGF-β2, TGF-β3, TGFβ-RI, and TGF-βRII mRNA. The expression levels of both TGF-β1 and TGF-β2 significantly reduced in the Tranilast (fold changes: 0.50 and 0.57) and TP (fold changes: 0.63 and 0.69) treated cells compared to the control. The combined treatment reduced the mRNA expression levels of TGF- β1 (fold change: 0.46) and TGF- β2 (fold change: 0.47), but they were higher than each one of the single treatments. TGFβ3 gene expression level was comparable in the TP treatment (fold change: 0.73), but it was significantly lower in Tranilast compared with the control (fold change: 0.64). Correspondingly, it also significantly decreased in the combined treatment group. The expressions of TGFβ-RI and TGFβ-RII genes significantly altered by both TP (TGFβ-RI fold change: 0.76 and TGFβ-RII fold change: 0.66) and Tranilast (TGFβ-RI fold change: 0.56 and TGFβ-RII fold change: 0.54) alone and also in the combination. Moreover, TGFβR3 expression level did not significantly decreased in the TP treatment group, but it significantly reduced in the Tranilast group (fold change: 0.79) and the combined treatment (fold change: 0.75) as compared to its levels in control cells (Figure 3). Additionally, Tranilast and TP reduced the expressions of the VEGFA gene (fold change: 0.47 and 0.62, respectively) in HUVECs. As well, the combination treatment highly decreased VEGFA expression (fold change: 0.42) (Figure 2). 

*The effects of TP, Tranilast, and their combination on rat’s microvessel outgrowth*

**Figure 4:**
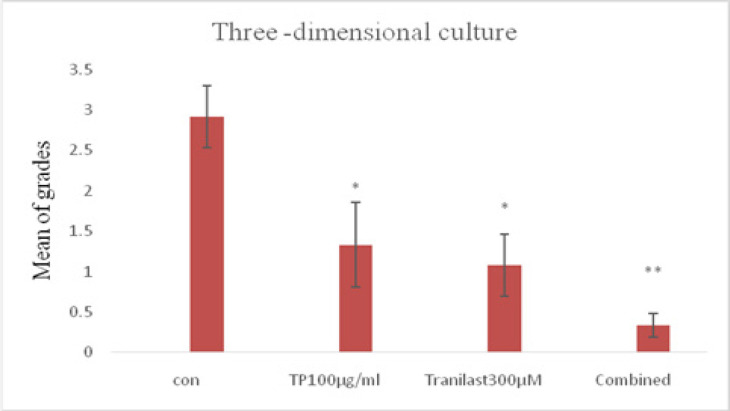
The Mean Score Obtained in the Control, 300μM Tranilast, 100 μg/ml TP, and Combined Groups; (*P < 0.05; ** P < 0.001 compared with the control)

Microscopic findings revealed vessel outgrowth raised from the rings into fibrin matrix on the day 10 of culturing. To determine whether the treatments could affect neovessel outgrowth, aorta rings were treated with 300 μM Tranilast, 100 μg/ml TP or their combination. According to the scoring system, the mean ±SD of the calculated angiogenesis response was estimated as 2.91± 0.38 in the control group, 1.08 ± 0.38 in the 300 μM Tranilast, 1.33 ± 0.52 in the 100 μg/ml TP, and 0.33 ± 0.14 in the combined group. It was observed that the combination of TP and Tranilast significantly inhibited angiogenesis (p<0.001) more than the administration of TP or Tranilast alone (p<0.05) (Figures 3 and 4).

**Table 1:** Primer Sequences Utilized for Real-Time PCR

Gene	Accession Number	Annealing temperature, °C	Size (bp)	Primer sequence
*ACTB*	NM_001101	60	146	F: 5ˊ-TGACCCAGATCATGTTTGAGACC-3ˊ
				R: 5ˊ-CTCGTAGATGGGCACAGTGTGGG-3ˊ
*VEGFA*	NM_001025366	60	105	F: 5ˊ-ATTATGCGGATCAAACCT-3ˊ
				R: 5ˊ-TTCTTGTCTTGCTCTATCTT-3ˊ
*TGFB1*	NM_000660	60	218	F: 5ˊ-AAGTGGACATCAACGGGTTC-3ˊ
	NM_000661			R: 5ˊ-GTCCTTGCGGAAGTCAATGT-3ˊ
*TGFB2*	NM_001135599	60	154	F: 5ˊ-CCATCCCGCCCACTTTCTAC-3ˊ
				R: 5ˊ-AGCTCAATCCGTTGTTCAGGC-3ˊ
*TGFB3*	NM_003239	60	201	F: 5ˊ-CAAAGGCGTGGACAATGAG-3ˊ
				R: 5ˊ-ACACAGCAGTTCTCCTCC-3ˊ
*TGFBR1*	NM_001306210	60	138	F: 5ˊ-ACATGATTCAGCCACAGATACC-3ˊ
				R: 5ˊ-GCATAGATGTCAGCACGTTTG-3ˊ
*TGFBR2*	NM_001024847	60	132	F: 5ˊ-GTAGCTCTGATGAGTGCAATGAC-3ˊ
				R: 5ˊ-CAGATATGGCAACTCCCAGTG-3ˊ
*TGFBR3*	NM_003243	60	170	F: 5ˊ-CCTTCCGTTTCCTTTCCCAGA-3ˊ
				R: 5ˊ-CACATTTGACAGACAGGGCAAT-3ˊ

## Discussion

Recently, we have reported that the combined treatment of HUVECs with TP and Tranilast could exert a synergistic effect on the proliferation capacity of HUVEC cells examined by MTT assay. In this regard, the combination index value estimated between 0.71 and 0.21 confirmed the synergistic effects of TP and Tranilast (Sheikhbahaei et al., 2018) .In the present study, trypan blue staining test also demonstrated that the HUVECs responded to the cytotoxic effects of the administered drugs both dose- and time-dependently. The combined treatment of the cells with these two drugs consequently reduced cell viability much higher than that of the TP and Tranilast alone. The most anti-proliferative properties of TP can be attributed to flavonoids and terpenoids. Components in TP influence variety of cell functions, which eventually result in the inhibition of cell proliferation (STANKOVIĆ et al., 2015). On the other hand, Tranilast decreases growth and differentiation of some cancerous cells, including prostate cancer cells (CU 145, PC3, (LNcap) (Murahashi et al., 1998) by increasing phosphorylation of AKT1 and decreasing the phosphorylation of ERK1 / 2 as well as G1/S cell cycle arrest in murine breast cancer cell line (Subramaniam et al., 2010). Our results are consistent with those of a previous study in which combined treatment with TP/vinblastine has decreased the survival rate of human cancer cell lines such as A431 MCF7, Skmel3, SW480, and EJ(Rajabalian, 2008). Angiogenesis is regulated through growth factors, cytokines, and chemokines; the most important of which are VEGFA , TGF-α, TGF-β, and bFGF (Ferrari et al., 2009). Accordingly, VEGFA induces endothelial cells proliferation, sprouting, and tubal formation under both in vivo and in vitro conditions (Otrock et al., 2007). It has been proved that TGF-β induces angiogenesis by regulating VEGFA expression for the spread and tumor metastasis, which is, in part, inhibited by Tranilast (Isaji et al., 1997). In the current study, we showed that Tranilast, TP, and their combination have the ability of decreasing VEGFA gene expression level in HUVECs, which results in the inhibition of angiogenesis. (Isaji et al., 1997). Koyama et al. in their study showed that Tranilast significantly inhibited VEGFA and bFGF in a dose-dependent manner (Koyama et al., 1999). In mammals, TGFβ has three isoforms (namely TGFβ1, β2, and β3) as well as three receptor isoforms (namely TGFβ-RI, TGFβ-RII, and TGFβ-RIII). Some researchers have revealed that TGFβ pathway plays a complex role in carcinogenesis, so that it performs both stimulatory and inhibitory effects on tumor vessel growth (Pardali et al., 2010). For instance, it was demonstrated that the TGF-β signaling pathway acts as a tumor suppressor at early stage of mammary carcinogenesis(Elliott and Blobe, 2005), while at later stages, as tumor progresses, levels of TGF-β increase and then it acts as a breast cancer stimulator (Walker and Dearing, 1992). In this regard, both TGF-β1 and TGF-βRII as important signaling pathways in angiogenesis, have been reported as the fundamental molecules for both vasculogenesis and Angiogenesis (Goumans et al., 2003). The use of a neutralizing antibody against TGF-β has also been proposed to inhibit angiogenesis (Duff et al., 2003).

The present study showed that the exposure of HUVECs to TP and Tranilast alone or in combination decreases the expression levels of TGF-β1, TGF- β2, TGF-β3, TGFβ-RI, and TGFβ-RII mRNA. However, it did not significantly decrease TGFβ3 and TGFβ-RIII following the TP treatment. Our findings are consistent with the results of a study by Izumi et al. (2009), who showed a dose-dependent inhibitory effect of Tranilast on TGFβ secretion in PC3 and Saos-2 cell lines (Izumi et al., 2009).

In another study, Yamamoto et al., (2009) investigated the effect of Tranilast on the growth of neurofibroma cells. Correspondingly, Tranilast inhibited the TGFβ secretion from fibroblasts, which plays an essential role in the treatment of keloids and hypertrophic scars (Yamamoto et al., 2009). Although the combination of Tranilast and tamoxifen has also decreased the expression levels of TGFβ1, TGF-β2, TGF-β3 TGFβ-RI, and TGFβ-RII, it increased the level of TGFβ-RIII in breast cancer cells (MCF-7) (Darakhshan and Ghanbari, 2013). 

 Ex-vivo aortic ring model could put the advantages of both in vivo and in vitro assays together and then bridges the gap between these two models. Of note, it was demonstrated that Neovessels growth occurs in a determined environment, and the culture system can be easily adapted to different experimental conditions (Nicosia and Villaschi, 1995).

 In vitro angiogenesis assays include proliferation, migration, and the formation of tube-like structures by endothelial cells (Park et al., 2016). According to our recent study on TP and/or Tranilast, as a result, these induced anti-angiogenic effects on HUVECs through cell viability alteration, a decrease in migration capacity, and an increase in the apoptosis of these cells (Sheikhbahaei et al., 2018). In the present study, we evaluated the angiogenic response of rat aortic rings to the administrations of TP and Tranilast alone and in combination. As well, the aortic outgrowth was evaluated by scoring new micro-vessels every 3 days for a 10-day duration. Subsequently, the treatment of rat aortic rings by TP and Tranilast led to a sharp decrease in vessels’ formation and extension. Moreover, TP has shown some antiproliferative effects on some cancer cell lines (Khazaei et al., 2018), which is a property that could be related to the anti-angiogenic effects of TP on HUVECs, as well. (Dehghan et al., 2016). The treatment of rat aortic rings with different doses of Eugenol has also resulted in the decreased new vessel formation and vascular sprouts extension after 48 h (Kouhestanian et al., 2015). However, other angiogenesis models such as chick chorioalantoic membrane and tumor inoculation, in an animal model like mice could strengthen our outcomes before coming to a reliable conclusion on the application of the TP and Tranilast combined treatment in human beings. 

In conclusion, the results of the present study clearly demonstrated that the combination of TP and Tranilast can provide a more significant anti-angiogenic effect compared with a single application in in vitro angiogenesis (HUVECs) as well as in ex vivo model of angiogenesis. However, further animal studies and clinical trials are required to evaluate the inhibitory therapeutic potential of the TP and Tranilast combination in the treatment strategies related to hyper-angiogenic diseases.

## Author Contribution Statement

F. Sheikhbahaei, SN. Nematollahi-Mahani and M. Khazaei were involved in experimental designs and carrying out the experiments. F. Sheikhbahaei and SN. Nematollahi-Mahani drafted the original manuscript. MR. Khazaei and S. Khazayel collected and analyzed the data. All authors approved the final version of the manuscript.
